# Janus porous polylactic acid membranes with versatile metal–phenolic interface for biomimetic periodontal bone regeneration

**DOI:** 10.1038/s41536-023-00305-3

**Published:** 2023-06-03

**Authors:** Yaping Zhang, Yi Chen, Tian Ding, Yandi Zhang, Daiwei Yang, Yajun Zhao, Jin Liu, Baojin Ma, Alberto Bianco, Shaohua Ge, Jianhua Li

**Affiliations:** 1 Department of Biomaterials, School and Hospital of Stomatology, Cheeloo College of Medicine, Shandong University & Shandong Key Laboratory of Oral Tissue Regeneration & Shandong Engineering Laboratory for Dental Materials and Oral Tissue Regeneration & Shandong Provincial Clinical Research Center for Oral Diseases, Jinan, China; 2 Department of Orthodontics, The First Affiliated Hospital of Zhengzhou University, (Stomatological Hospital of Henan Province), Zhengzhou, China; 3 CNRS, Immunology, Immunopathology and Therapeutic Chemistry, UPR 3572, University of Strasbourg, ISIS, Strasbourg, France

**Keywords:** Periodontitis, Implants, Tissue engineering

## Abstract

Conventional treatment to periodontal and many other bone defects requires the use of barrier membranes to guided tissue regeneration (GTR) and guided bone regeneration (GBR). However, current barrier membranes normally lack of the ability to actively regulate the bone repairing process. Herein, we proposed a biomimetic bone tissue engineering strategy enabled by a new type of Janus porous polylactic acid membrane (PLAM), which was fabricated by combining unidirectional evaporation-induced pore formation with subsequent self-assembly of a bioactive metal–phenolic network (MPN) nanointerface. The prepared PLAM-MPN simultaneously possesses barrier function on the dense side and bone-forming function on the porous side. In vitro, the presence of MPN nanointerface potently alleviated the proinflammatory polarization of mice bone marrow-derived macrophages (BMDMs), induced angiogenesis of human umbilical vein endothelial cells (HUVECs), and enhanced the attachment, migration and osteogenic differentiation of human periodontal ligament stem cells (hPDLSCs). The implantation of PLAM-MPN into rat periodontal bone defects remarkably enhanced bone regeneration. This bioactive MPN nanointerface within a Janus porous membrane possesses versatile capacities to regulate cell physiology favoring bone regeneration, demonstrating great potential as GTR and GBR membranes for clinical applications.

## Introduction

Periodontal bone defect caused by periodontitis, tumor or trauma can damage the integrity of periodontal tissue, affecting the aesthetics, occlusal function and quality of life of patients. The repair of theses defects and the regeneration of bone still remain major clinical challenges^1^. In recent years, guided tissue engineering (GTR) and guided bone regeneration (GBR) have emerged as important strategies to address these challenges, both of which involve the restoration of periodontium/bone defects by mean of barrier membranes^2,3^. Bone substitute materials are commonly used in combination with the barrier membrane during surgical implementation as the traditional barrier membrane does not have enough capability for bone regeneration, which is both time-consuming and costly for clinical practice^4–6^.

The natural biological process of bone regeneration includes immune cell response during the initial inflammatory stage, followed by endothelial vascular neogenesis and subsequent stem cell recruitment and osteogenic differentiation during the remodeling stage^7^. Therefore, an ideal GTR/GBR membrane is expected to possess superior capacities to actively regulate these bone-repair-related cells (e.g. immune cells, endothelial cells and stem cells), while avoiding faster-growing non-osteogenic fibrous cells or tissues from migrating into the defect to interfere with the bone regeneration process.

To realize these goals, numerous membranes with a bi-layered or “Janus” structure have been developed, including electrospun nanofiber double-layer membranes, layer-by-layer assembled polymer/ceramic composites, and 3D-printed scaffolds with gradient porosities^8–11^. In these studies, low-cost and easy-processed polymers such as polylactic acid (PLA) have been widely used^8^. Nevertheless, since synthetic polymer substrates are normally lacking of bioactive sides to interact with bone-repair-related cells^12,13^, a simple bioactive polymer membrane with anisotropic structures for bone repair is highly demanded.

Assembly of nanostructured interfaces on biomaterials is proven to be an effective way for manipulating cell behavior to facilitate tissue regeneration^14^. Metal phenolic network (MPN), a complex supramolecular structure formed by the coordination of metal ions and phenolic ligands, has been widely studied as bioactive nanocoating for implant modification, drug release and stem cell differentiation^15–17^. It is reported that numerous naturally-derived phenolic molecules (e.g. tannic acid (TA) and catechins) are beneficial for anti-inflammatory and bone forming purposes^18,19^. Metal ions such as copper ions (Cu^2+^), a trace element in the human body, have been considered to promote tissue healing by stimulating angiogenesis and stem cell differentiation^20,21^. In addition, MPN is biodegradable in physiological environment^22–24^, therefore these therapeutic metal ions and phenolic ligands can be disassembled and slowly released to the defect tissue. Therefore, we hypothesized that introducing TA/Cu^2+^-based MPN nanocoating to a Janus porous membrane could provide a bioactive interface for immunomodulation, angiogenesis, stem cell regulation, that may achieve a biomimetic bone regeneration.

**Fig. 1:**
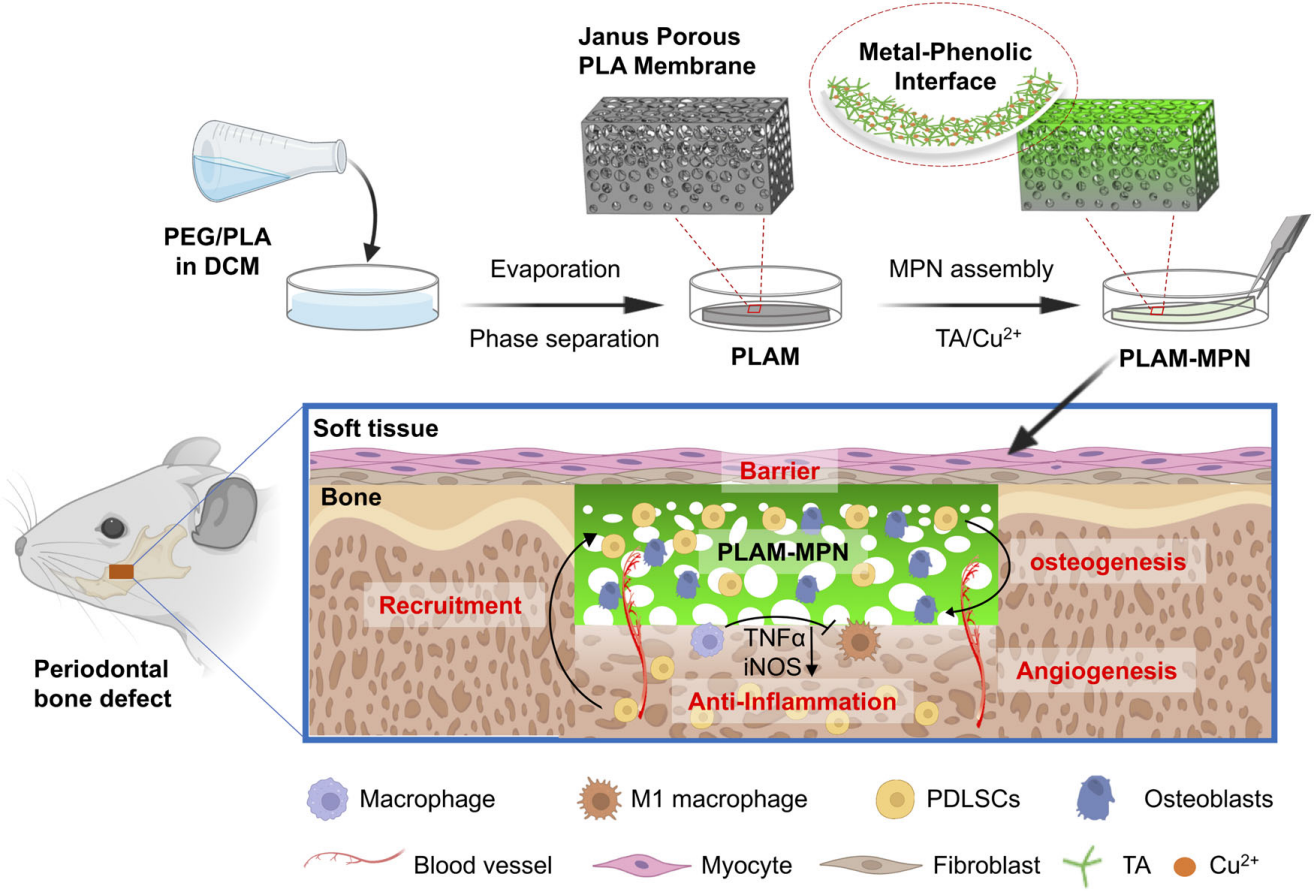
The design and working mechanism of the Janus porous PLA membrane with MPN nanointerface for biomimetic bone regeneration.

Herein, we reported a Janus porous PLA membrane (PLAM) modified with MPN nanointerface (PLAM-MPN) that was fabricated by combining unidirectional evaporation-induced pore formation with subsequent interfacial self-assembly of TA and Cu^2+^. We envisioned that the acquired PLAM-MPN could possess barrier function on the dense side and bone-forming function on the MPN-coated porous side (Fig. 1). To test the hypothesis, we examined the ability of PLAM with different MPN nanointerface coatings to regulate bone-repair-related cells by studying their interactions with rat bone marrow-derived macrophages (BMDMs), human umbilical vein endothelial cells (HUVECs), and human periodontal ligament stem cells (hPDLSCs) in vitro. Furthermore, implantation of PLAM-MPN into a rat periodontal defect model was performed to investigate their potential for biomimetic periodontal bone regeneration.

## Results

### Characterization of the Janus porous membrane

**Fig. 2:**
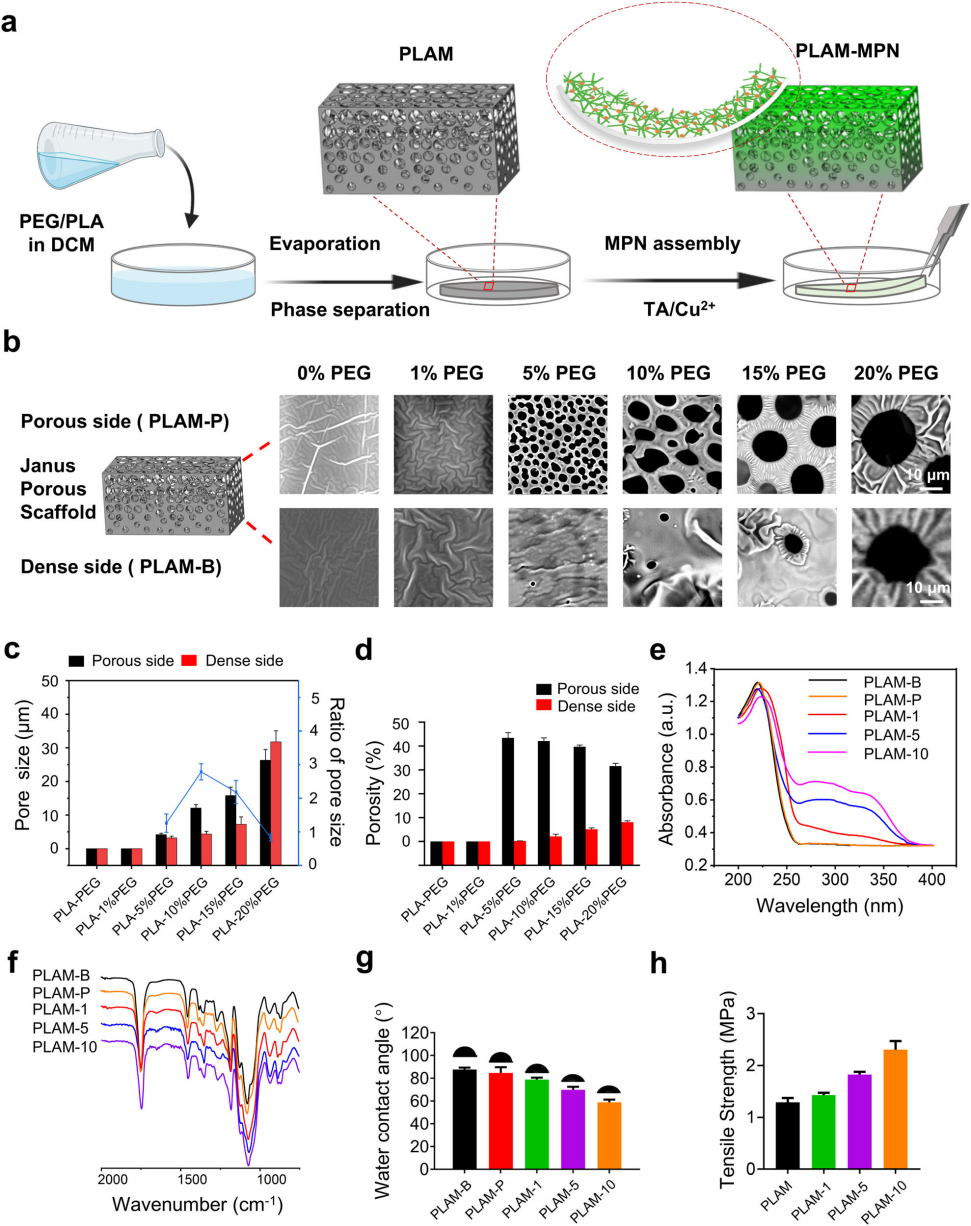
Physicochemical structure characterization of the scaffolds. **a** Schematic illustration of the preparation process of Janus porous PLAM and self-assembly of MPN nanointerface. **b** SEM images of the Janus porous structure of PLAM scaffold (upper row: PLAM-P; lower row: PLAM-B) prepared with different PEG contents. **c** Average pore size of porous side and dense side, and the ratio of pore size of the porous side and the dense side. **d** Porosity of porous side and dense side. **e** WCA hydrophilicity measurement. **f** UV-vis absorption spectra. **g** FTIR spectra and **h** mechanical test of the pristine PLAM and PLAM-MPN scaffolds. *n* = 3 independent experiments, all data are shown as mean ± SD.

The manufacturing process of the PLAM scaffold with the assembled MPN nanointerface is shown in Fig. 2a. The morphology of the PLAM was characterized by SEM (Fig. 2b), one side of the PLAM possessed a porous structure (denoted as PLAM-P), while the other side of the membrane showed a dense barrier structure (denoted as PLAM-B). As shown in Fig. 2c, d, the pore size and porosity of the pristine PLAM could be adjusted by the amount of added PEG^25^. As PEG content was increased, the pore size of PLAM-P or PLAM-B side increased correspondingly, reaching the maximum value at 20% PEG. However, the ratio between the pore sizes in the two sides of the membrane decreased when the PEG content was higher than 10%, in favor of bigger pores in PLAM-B part. Considering that the pore size and porosity of the PLAM-P side is expected to be large enough to be conducive to the ingrowth of bone cells and that the PLAM-B side needs to be dense with small pore size and less porosity to act as a barrier, pristine PLAM prepared at 15% PEG with larger pore size was finally chosen for subsequent experiments.

Before harvesting the Janus porous PLAM from the drying plate, the pristine membranes were incubated with Cu^2+^ and TA solution to initiate the MPN nanofilm assembly on the porous side to obtain the final PLAM-MPN with 1, 5, 10 MPN layers (PLAM-1, 5, 10). Figure 2e shows the UV–vis absorption spectra of PLAM-MPN scaffolds, where the strong peak at 260-340 nm was attributed to the absorption caused by the chemical coordination of Cu-TA^26^. The FTIR spectra of PLAM-MPN (Fig. 2f) evidenced the strong peak at 1759 cm^−1^ attributed to the absorption of the ester group and the absorption peaks at 1690–1500 cm^−1^ attributed to the C = C bending vibration of TA, respectively^27^.

The surface wettability of a scaffold is an important parameter affecting the overall performance of cell growth in tissue engineering^28^. The wettability of the scaffolds was evaluated by water contact angle (WCA) measurements (Fig. 2g), the contact angle of the PLAM-10 scaffolds significantly reduced to 56.4°, while the contact angle of PLAM-B was as high as 89.0°. The anisotropic difference of hydrophilicity in PLAM could favor its GTR function by affecting cell attachment. Moreover, the bulk elastic modulus of the PLAM-MPN scaffolds significantly increased compared to that of PLAM (Fig. 2h). The mechanical enhancement could ensure that the scaffolds provide a sufficient mechanical support for bone tissue regeneration.

The above results indicated the successful assembly of MPN nanointerface on the Janus porous PLAMs, endowing the PLAM-MPN scaffolds with anisotropic surface property and enhanced mechanical properties.

### Stem cell viability, recruitment and attachment on the MPN nanointerface

**Fig. 3:**
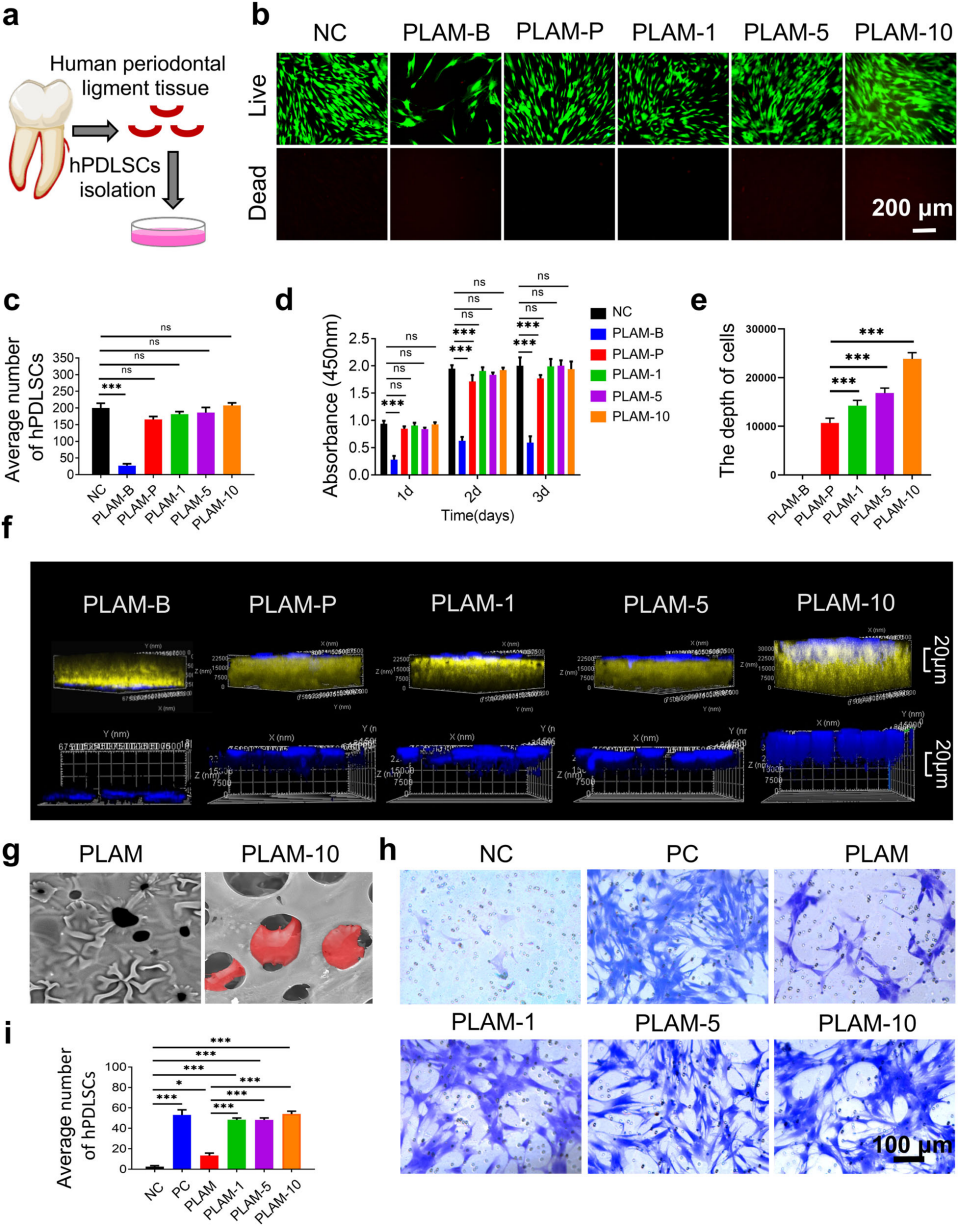
In vitro study of cell attachment, viability, infiltration and migration of hPDLSCs on the PLAM and PLAM-MPN scaffolds. **a** The isolation and culture of hPDLSCs. **b** Representative images and **c** quantitative analysis of Live/Dead staining of hPDLSCs incubated on the different scaffolds for 48 h (*n* = 3 independent experiments, four random fields in each sample, scale bar = 200 μm). **d** CCK-8 assay at 24, 48, 72 h (*n* = 3 independent experiments). **e**, **f** Depth of hPDLSCs entry into the scaffolds and representative CLSM images of hPDLSCs in different groups at 48 h (*n* = 3 independent experiments, four random fields in each sample, scale bar = 20 μm). **g** SEM of hPDLSCs in the PLAM scaffold and PLAM-10 scaffold. Red area indicated the cell infiltrated inside the scaffold. (*n* = 3 independent experiments, scale bar = 10 μm). **h**, **i** Representative crystal violet staining images and quantitative analysis of hPDLSCs recruited on the scaffolds. NC, 0.1% FBS. PC, 10% FBS (*n* = 3 independent experiments, four random fields in each sample, scale bar = 100 μm). All data are shown as mean ± SD, ^*^P < 0.05, ^**^P < 0.01 and ^***^P < 0.001.

The periodontal ligament stem cells are the main repairing cells in the repair of periodontal defects as they have self-renewal and multi-directional differentiation potential^29^. To investigate the biocompatibility of the MPN nanointerface, hPDLSCs isolated from human periodontal ligament tissues (Fig. 3a) were cultured with different membrane samples. Live/dead staining results showed that there was no statistical difference among all groups except that PLAM-B decreased the number of hPDLSCs adhered to the scaffolds, indicating that the porous side of PLAM facilitated stem cell adhesion and proliferation, and the dense barrier side was not conducive to the attachment and growth of the cells and could be utilized as a barrier membrane (Fig. 3b, c). Meanwhile, CCK-8 assay results were consistent with the Live/Dead staining (Fig. 3d). The cell viability on each sample increased with prolonged culture time from 1 to 3 d, indicating that prepared membranes facilitated stem cell proliferation.

In addition, CLSM was used to observe the 3D attachment of hPDLSCs on each sample. Except for PLAM-B group, hPDLSCs well adhered onto the PLAM-P side of the scaffolds. Moreover, the cell penetration deepened with the increased thickness of MPN (Fig. 3e, f), because the MPN coating is likely favorable for cell migration^16,30^. The detailed cell morphologies on the PLAM-10 could be clearly observed by SEM imaging, showing that the stem cell grew inside the pores and the cell body was well spread on the pore surface (Fig. 3g).

The enhanced cell migration capacity of hPDLSCs in the presence of the scaffolds was then investigated by performing the transwell migration experiment. As shown in Fig. 3h, i, compared with the NC and PLAM scaffolds, the PLAM-10 scaffold significantly increased the number of migrated cells. The results indicate that MPN coating could stimulate cell migration in vitro, which is possibly due to the characteristics that TA-Cu MPN nanocoating is biodegradable and the release of Cu^2+^ can mediate the migration of hPDLSCs through the activated hypoxia inducible factor-1α (HIF-1α) pathway and the upregulation of chemokine receptor Rnd3^30^. Therefore, the PLAM-10 scaffold is expected to recruit stem cells to the bone defect side to promote tissue regeneration in vivo.

### In vitro immunoregulation of PLAM-MPN

**Fig. 4:**
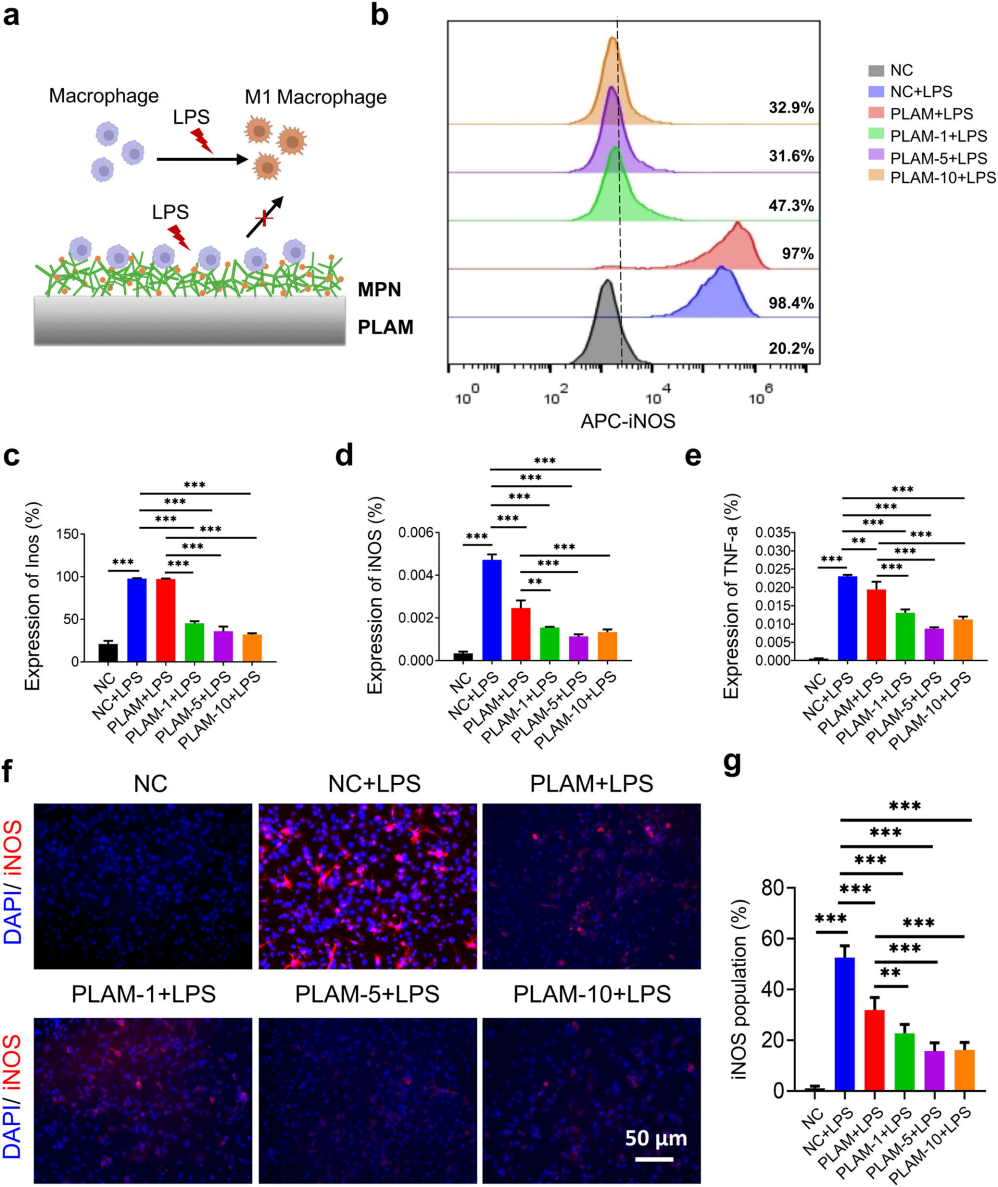
In vitro immunoregulation of the BMDMs on different scaffolds. **a** Schematic illustration of biomaterial-mediated macrophage polarization. **b**, **c** Flow cytometry assay and quantitative analysis of iNOS in BMDMs stimulated with LPS for 24 h (*n* = 3 independent experiments). **d**, **e** Relative mRNA expressions of iNOS and TNF-α in BMDMs stimulated with LPS for 24 h. **f**, **g** Representative immunofluorescent staining images (blue represents DAPI; red represents iNOS) and quantitative analysis of iNOS in BMDMs stimulated with LPS for 24 h (*n* = 3 independent experiments, four random fields in each sample, scale bar=50 μm). All data are shown as mean ± SD, ^**^P < 0.01 and ^***^P < 0.001.

Immune regulation plays an important role in the osteogenesis process of bone defects, and the polarization and secretion of macrophages are particularly critical^31,32^. In order to explore the regulatory effect of these scaffolds, BMDMs (95.6% purity, Supplementary Fig. 1) were co-cultured with each sample and their polarization behavior was analyzed by flow cytometry, qRT-PCR and immunofluorescence staining (Fig. 4a). The results of flow cytometry showed that the BMDMs treated with LPS were significantly skewed towards the iNOS^+^ M1 subpopulation, while the cells treated with the PLAM-MPN scaffolds significantly dampened M1 polarization (Fig. 4b, c). The gating strategy used to identify M1-type macrophages is outlined in Supplementary Fig. 2. The results of qRT-PCR (Fig. 4d, e) showed that the PLAM-MPN scaffolds significantly down-regulated the expression levels of M1 type-related genes, including *iNOS* and *TNF-α*. Meanwhile, there was no statistical difference in the expression of M2 subsets or M2-related *CD206* and Arg-1 genes among the PLAM-MPN groups (Supplementary Fig. 3), and Supplementary Fig. 4 Shown the gating strategy for M2-type macrophages. The immunofluorescence staining (Fig. 4f, g) of iNOS in BMDMs further confirmed the immunoregulatory ability of PLAM-MPN, which effectively reduced the inflammatory response by inhibiting the polarization of macrophages towards M1 type and the expression of pro-inflammatory factors, which may be related to the inhibition of macrophage TLR4 signaling pathway by TA in the MPN coating^18^.

### In vitro vascularization of endothelial cells on the MPN nanointerface

**Fig. 5:**
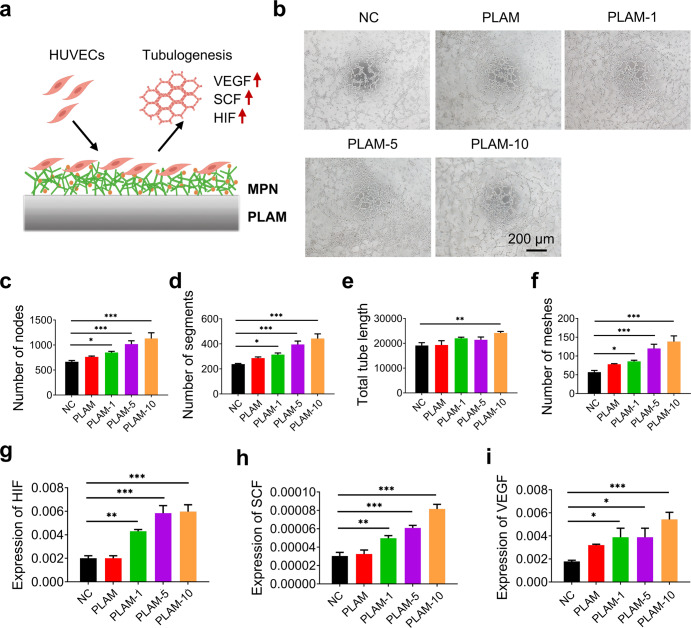
In vitro angiogenesis of HUVECs on different scaffolds. **a** Schematic illustration of biomaterial-mediated tubule formation. **b** Representative optical images of tube formation of HUVECs in different groups cultured for 14 d (*n* = 3 independent experiments, four random fields in each sample, scale bar = 200 μm). **c**–**f** Quantitative analysis of number of nodes, segments, meshes and total tube length of the formed tubules. **g**–**i** qRT-PCR analysis for the relative mRNA levels of angiogenesis-related gene HIF, SCF and VEGF (*n* = 3 independent experiments). All data are shown as mean ± SD, ^*^P < 0.05, ^**^P < 0.01 and ^***^P < 0.001.

Angiogenesis is an integral step during regeneration process as the vascular network formation are critical for providing nutrition for the repairing cells. In this context, vascular endothelial cells play a crucial role in maintaining the physiological functions of developing blood vessels^33^. Therefore, HUVECs were used in vitro to characterize angiogenesis inducing ability of MPN nanointerface by tubule formation assay and qRT-PCR analysis (Fig. 5a). As shown in Fig. 5b–f, the PLAM-10 group exhibited stronger angiogenic capacity, and higher node counts, segments, meshes, and total tube length compared to the control and PLAM groups. In addition, the expressions of angiogenic genes such as HIF, SCF and VEGF^34–36^ were further assessed by qRT-PCR. Compared with other groups, the relative expression levels of angiogenic genes were significantly up-regulated in PLAM-10 group (Fig. 5g–i). These results indicated that the MPN nanointerface in PLAM-10 membrane had a strong induction effect on the angiogenesis of HUVECs, which is beneficial for periodontal bone regeneration.

### In vitro stem cell osteoinductive ability of the PLAM-MPN

**Fig. 6:**
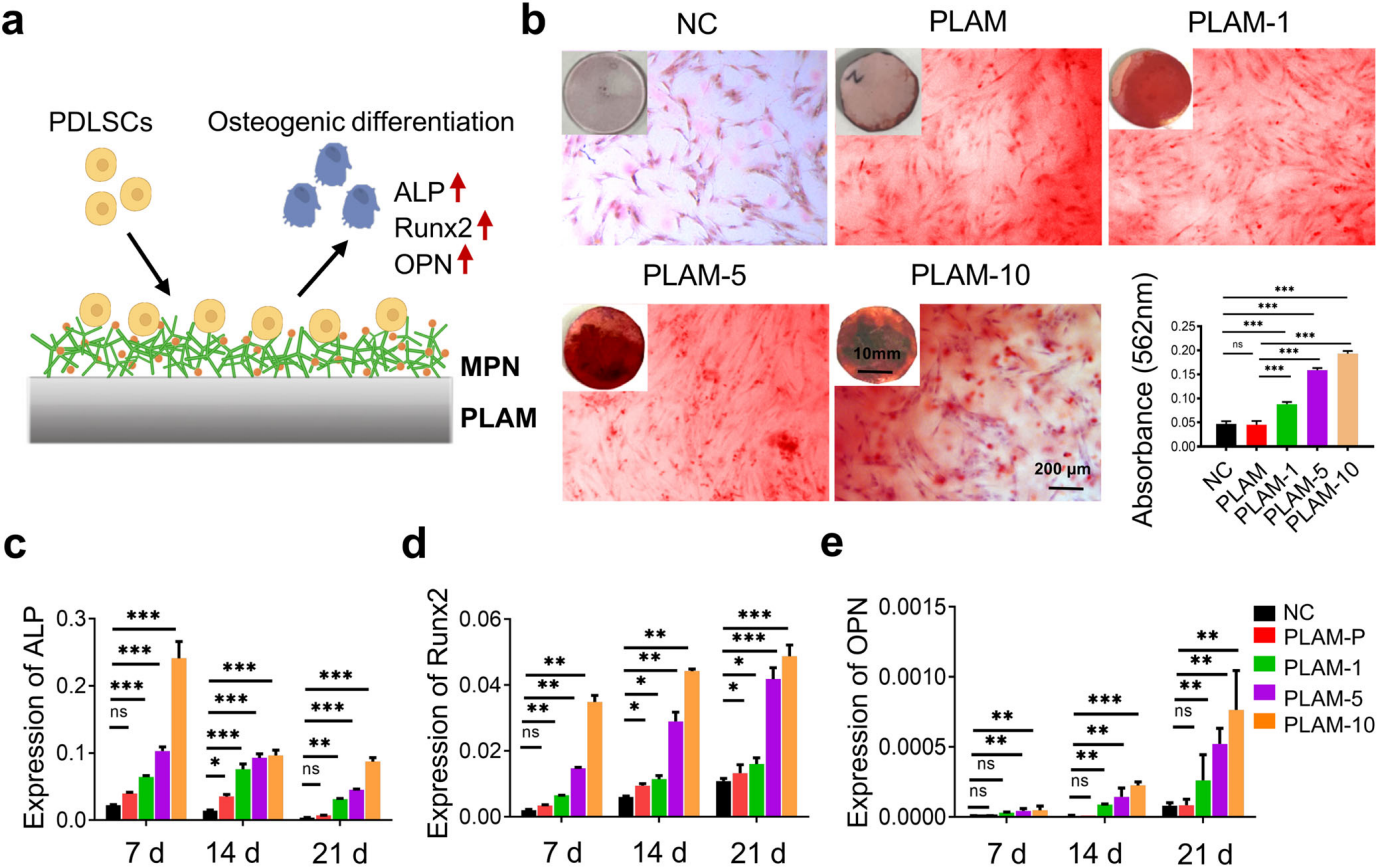
In vitro osteogenic differentiation of hPDLSCs on different scaffolds. **a** Schematic illustration of biomaterial-mediated osteogenic differentiation. **b** Representative photographs of Alizarin red staining of hPDLSCs and quantitative analysis of mineralized nodules in different groups at 21 d (macrographs of stained cells were inserted, *n* = 3 independent experiments, four random fields in each sample, scale bar = 200 μm). **c**–**e** qRT-PCR analyses for the relative mRNA levels of osteogenesis-related genes of ALP, Runx2, and OPN at 7, 14, and 21 d (*n* = 3 independent experiments). All data are shown as mean ± SD, ^*^P < 0.05, ^**^P < 0.01, and ^***^P < 0.001.

An ideal material for repairing periodontal bone defects should have the ability to enhance the osteogenic differentiation of hPDLSCs^29,37^. The mineralized nodule formation ability of hPDLSCs was detected by Alizarin red staining and CPC colorimetry after co-culturing these cells with the different scaffolds for 21 d (Fig. 6a). Alizarin red staining of the calcified extracellular matrix clearly demonstrated the enhanced mineralized nodule formation of hPDLSCs cultured on the porous side of membrane with MPN coating (Fig. 6b). The results of calcium mineral deposition measured by CPC colorimetry further quantitatively confirmed the osteoinductive effect of MPN with nearly 3-fold of increase (Fig. 6b).

In addition, the gene expression levels of osteogenic markers, including ALP, Runx2 and OPN, after osteogenic induction for 7, 14, and 21 d were detected by qRT-PCR, respectively. ALP is an early marker of osteoblast differentiation and plays a key role in calcification in vitro^38^. As shown in Fig. 6c, ALP in PLAM-10 group was significantly up-regulated by more than 5-fold compared with the NC group at 7 d. Runx2 is an essential transcription factor for osteoblast differentiation, which could promote bone formation and inhibit bone resorption^39^. The expression level of Runx2 in PLAM-10 group was significantly increased at 7 d compared with other groups, and continued to increase at 14 and 21 d (Fig. 6d). OPN is a matrix protein secreted by osteoblasts and plays an important role in bone regeneration^40^. The expression level of OPN in the PLAM-10 group gradually increased after induction, reaching the maximum level at 21 d, and was significantly higher than that in other groups (Fig. 6e).

The above results indicated that the MPN coating promoted osteogenic differentiation of hPDLSCs. Meanwhile, the expression of all osteogenic genes was not statistically different between the PLAM scaffold and the NC group, indicating that the pristine PLAM scaffold itself cannot regulate osteogenic differentiation, consistent with the results reported in Fig. 6d–f. The mechanism may be that Cu^2+^ up-regulates the expression of HIF-1α in bone marrow mesenchymal stem cells by activating the Erk1/2 signaling pathway^41,42^, resulting in the secretion of VEGF and BMP-2 proteins, thereby achieving the goal of promoting bone formation.

### In vivo bone regeneration of the PLAM-MPN

**Fig. 7:**
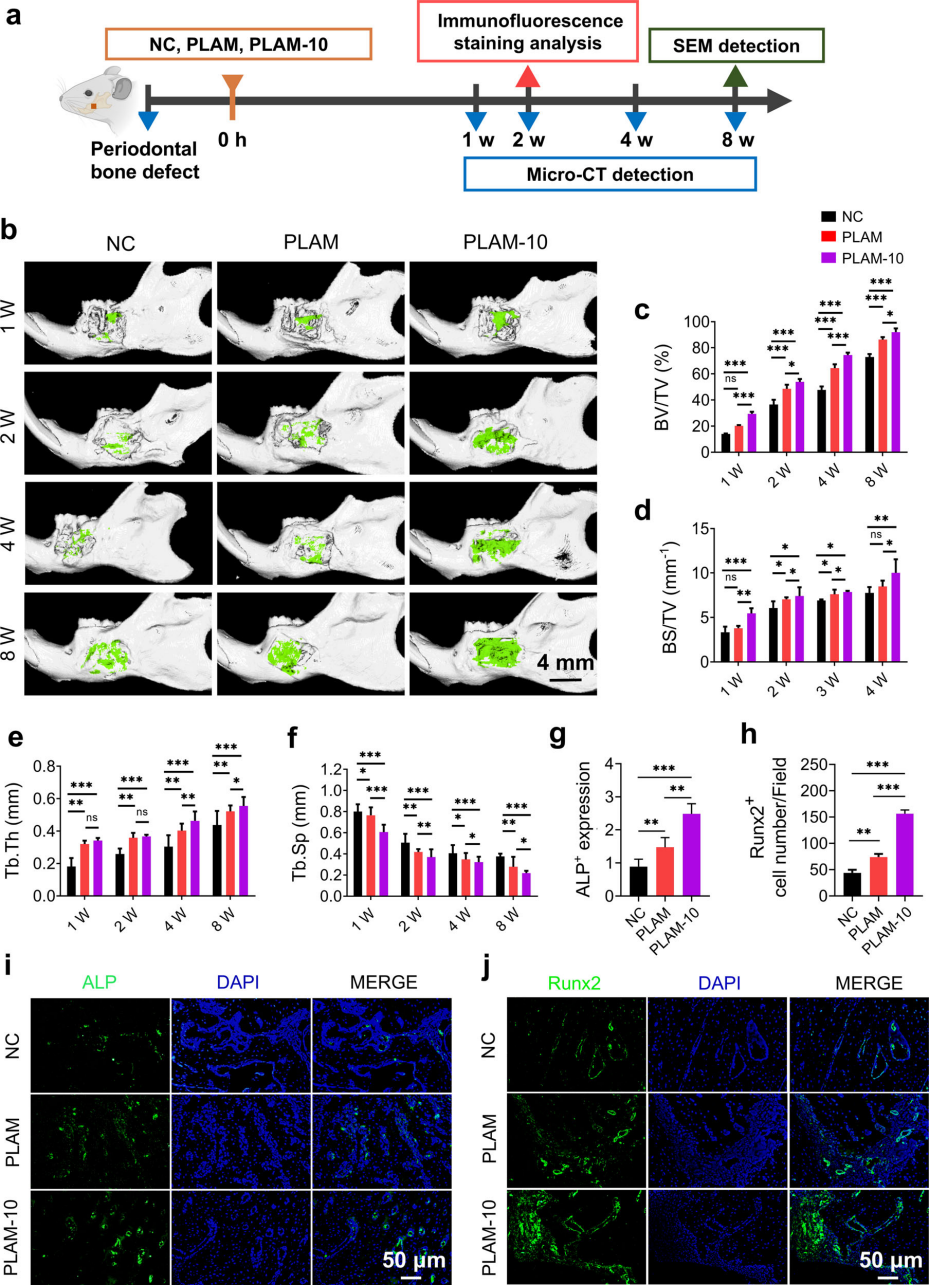
In vivo evaluation of the bone regeneration activity of the scaffolds in a rat periodontal bone defect model. **a** Schematic illustration. **b** Reconstructed Micro-CT images of bone defects at 1, 2, 4, and 8 w post-surgery, green represents nascent bone. Scale bar = 4 mm). **c**–**f** Quantitative analysis of BV/TV, BS/TV, Tb.Th and Tb.Sp (*n* = 6 independent experiments). Representative immunofluorescence staining images and quantitative analysis of mandibular bone defects at week 2 post-operation (*n* = 6 independent experiments, three random fields in each sample, scale bar=50 μm). **g**–**i** ALP staining (blue represents DAPI; green represents ALP), **h**–**j** Runx2 staining (blue represents DAPI; green represents Runx2). All data are shown as mean ± SD, ^*^P < 0.05, ^**^P < 0.01 and ^***^P < 0.001.

The above in vitro results revealed the barrier function of the Janus porous structure and the regulation of different bone repairing-related cells based on the MPN nanointerface. This motivated us to evaluate their in vivo biomimetic bone regeneration ability in a rat periodontal bone defect model (Fig. 7a). As shown by the Micro-CT results, the bone defect implanted with the PLAM-10 scaffolds were rich in more newly formed bone tissue compared with the other two groups (Fig. 7b). To represent new bone formation in a quantitative and qualitative way, we evaluated various bone mass-related parameters, including BV/TV and BS/TV^43,44^. As could be seen from the Fig. 7c, the BV/TV of the PLAM-MPN group was much higher than that of the other two groups at week 1, 2, and 4. Notably, the BV/TV of the PLAM-10 group was still higher than the other two groups at 8 w, which was consistent with the micro-CT images. The BS/TV values showed a similar trend, that is, the BS/TV values of the PLAM-10 group were significantly higher than those of the other two groups at 1, 2, 4, and 8 w after implantation (Fig. 7d). In addition, the Tb.Th of the PLAM-10 group was significantly higher than that of the other two groups at 2, 4 and 8 w (Fig. 7e). Conversely, Tb.Sp decreased over time, indicating that newly formed bone gradually became denser and more mature (Fig. 7f). In conclusion, the PLAM-10 scaffolds not only significantly improved bone mass (BV/TV and BS/TV), but also significantly promoted bone quality (Tb.Th and Tb.Sp) during the entire bone regeneration process. Moreover, there was no significant difference in the evaluation of micro-CT related parameters between the NC group and the PLAM group, indicating that the scaffolds alone were not able to promote bone regeneration, and the modification of the MPN active coating greatly improved the osteogenic effects in vivo periodontal bone defects. The results of immunofluorescence staining showed that the expression levels of ALP and Runx2 in bone defects in the PLAM-10 group were significantly higher than those in other groups (Fig. 7g–j), consistent with the in vitro study that PLAM-10 promoted the osteogenic differentiation of hPDLSCs. This study confirmed that PLAM-10 can promote the differentiation of hPDLSCs into osteoblasts by increasing the recruitment and attachment of hPDLSCs. Compared with PLAM, PLAM-10 not only enhanced bone mass, but also improves the quality of new bone, and successfully augmented the bone regeneration.

### In vivo evaluation of angiogenesis and immune regulation

**Fig. 8:**
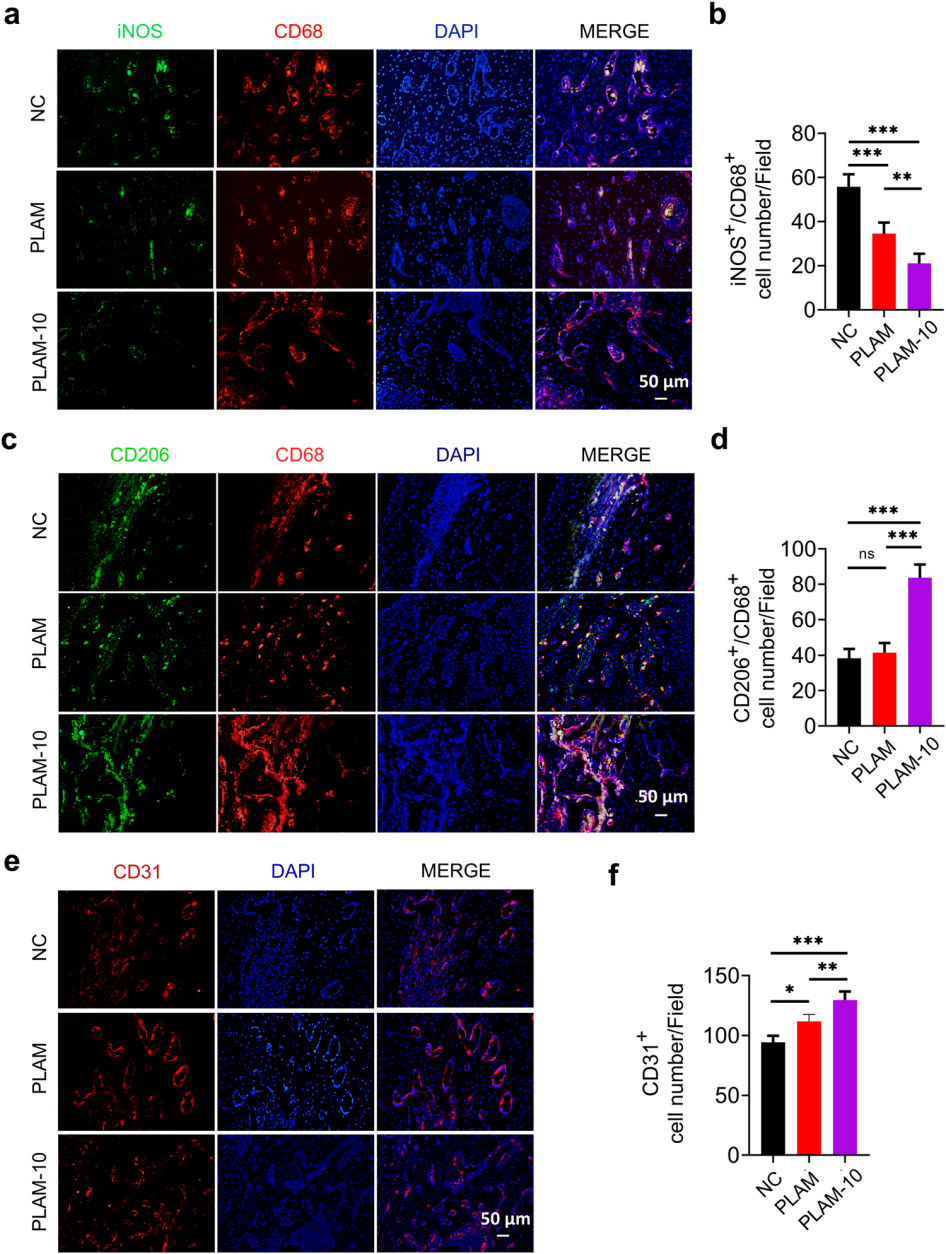
In vivo evaluation of the role of scaffolds in immune regulation and angiogenesis. Representative immunofluorescence staining images and quantitative analysis of bone defects at week 2 post-operation (*n* = 6 independent experiments, three random fields in each sample, scale bar = 50 μm). **a**, **b** iNOS, CD68 staining, **c**, **d** CD206, CD68 staining, **e**, **f** CD31 staining. Scale bar = 50 μm. All data are shown as mean ± SD, ^*^P < 0.05, ^**^P < 0.01 and ^***^P < 0.001.

Bone repair and regeneration are closely related to immune regulation and nutritional support^45^. In order to evaluate the regulatory effect of PLAM-10 on immune cells and angiogenesis in vivo, the related indexes in bone defect side were observed at 2 w after operation. The results of immunofluorescence staining showed that the number of iNOS^+^ cells at the bone defect side in the PLAM-10 group was significantly smaller than that in the other groups (Fig. 8a, b), while the proportion of CD206^+^ cells was higher (Fig. 8c, d), indicating that PLAM-10 inhibited the polarization of local macrophages towards pro-inflammatory M1 subtype and shifted the polarization towards anti-inflammatory M2, thereby regulating the local inflammatory microenvironment and facilitating tissue regeneration. At the same time, the number of CD31^+^ cells in the injury center of the PLAM-10 group significantly increased (Fig. 8e, f), which indicated that the scaffold material enhanced angiogenesis at the bone detect side.

Moreover, from the SEM imaging of the membranes taken out 8 w after surgery, it was observed that there was almost no infiltration of cells on the barrier surface of PLAM and plenty of cells infiltrated into the porous side (Supplementary Fig. 5), which also indicated that the Janus porous structure played as a good barrier function in the process of bone repairing. This membrane can block the infiltration of peripheral inflammatory cells and fast-growing soft tissue, which is conducive to regulate and improve the microenvironment of bone defect sides and promote repair.

## Discussion

The effective repair of periodontal bone defects is still a major clinical challenge. At the same time, the development of barrier membrane materials with barrier and bone regeneration-promoting functions is an important research direction towards periodontal tissue regeneration. In this study, a Janus porous PLAM-MPN scaffold was constructed, the dense side acting as a barrier, and the other side being porous as a scaffold for guiding bone regeneration. The MPN bioactive nanointerface was introduced in guided tissue regeneration membrane for the first time, and it was proved that the MPN nanocoating on PLAM significantly inhibited the pro-inflammatory state of macrophages, promoted angiogenesis of HUVECs and enhanced the osteogenesis of hPDLSCs. In a rat periodontal bone defect model, the PLAM-MPN barrier membrane facilitated the repair of periodontal bone defects by synergistically regulating the immune, angiogenesis, and osteogenic processes. Therefore, this Janus porous barrier membrane with an effective MPN bioactive nanointerface provides a powerful biomimetic regeneration strategy for realizing periodontal bone defect repair.

On the other hand, the Janus porous membrane based on FDA-approved PLA polymer has a simple preparation method, low cost of raw materials and easy large-scale processing. It should be noted that the use of PEG during processing may cause potential immunogenicity issues according to recent studies^46,47^, therefore alternative pore-forming techniques to fabricate Janus porous PLA need to be explored in future study to address the biosafety issue before clinical translations. Nevertheless, the MPN coating demonstrated in this paper can be considered a simple and general surface modification method for various substrates. Considering the wide variety of polyphenols and metal ions and their versatile biological activities, this strategy can be adopted to other barrier membranes as well as to many other forms of tissue engineering materials.

## Methods

### Fabrication of the scaffolds

PLAM were prepared by evaporative phase separation method. The preparation process was as follows: dichloromethane (DCM) was added to a glass bottle, and then polyethylene glycol (PEG) 200 was added to DCM, the mass ratio of PEG and DCM was 1:5. The mixture was vigorously stirred, then PLA (13.3% w/w DCM) was dispersed in the PEG solution, and the glass vial was sealed and placed under sonication at 37 °C to minimize solvent evaporation. The sonication was supplemented with stirring until the mixture was homogeneous. The mixture was poured over a glass dish to distribute evenly and the glass dish was then placed in a fume hood at room temperature for 24 h to remove residual solvent. Finally, by removing the thin film (300–500 µm in thickness) from the glass plate, the Janus porous barrier film was obtained.

### Fabrication of the MPN

MPN was obtained by a layer-by-layer method. The preparation process was as follows: the prepared PLAM scaffold in the plate was washed three times with deionized water. Next, the PLAM scaffold (100 mm in diameter) was soaked with 10 mL of TA solution (0.8 mg mL^−1^) at room temperature, shaken for 30 s at 75 rpm, followed by the addition of 10 mL of Cu^2+^ solution (0.2 mg mL^−1^), shaken again for 30 s at 75 rpm. Then, 100 μL of sodium hydroxide solution (pH = 13) was added to adjust the pH to 7.5 and shaken for 30 s at 75 rpm. Finally, the scaffold was washed thrice with deionized water to obtain the MPN-loaded PLAM scaffold. According to the number of MPN layers required subsequently, the above preparation process was repeated 1, 5, and 10 times to obtain PLAM-1MPN (PLAM-1), PLAM-5MPN (PLAM-5), and PLAM-10MPN (PLAM-10) scaffolds, respectively.

### Characterization of the scaffolds

The morphology of the scaffolds was observed using a scanning electron microscope (SEM) (Pro G5, Phenome) at an accelerating voltage of 5 kV. To corroborate the different chemical composition of the PLAM and the PLAM-MPN scaffolds, ultraviolet spectrophotometer (UV) (Shimadzu, UV2600 Ι) was used to detect the characteristic peak of Cu-TA. To detect whether Cu^2+^ was loaded successfully on the surface of the PLAM-MPN scaffolds, Fourier infrared spectrometer (FTIR) (Bruker, ALPHA II) was used to scan over the range of 50–3500 cm^−1^.

The surface wettability of the scaffolds was detected by static water contact angle measurement at room temperature (26 ± 1 °C). A contact angle instrument (Zhongchen, JC2000) was utilized, where 2.0 µL deionized water was automatically dropped onto the scaffold and recorded using a digital camera until the water droplet on the scaffold displayed a stable shape. Three points at least from different positions of each sample were recorded and averaged to determine the static water contact angle.

The tensile strength of scaffolds (10 × 50 mm) was characterized using a universal materials tester (Shimadzu, Japan). All samples were stretched at a constant tensile rate of 10 mm min^−1^ and the original length of the strips was 30 mm. Each sample was measured for three times in the tensile tests.

### Cell culture

The following study protocol was approved by the Medical Ethical Committee of School of Stomatology, Shandong University (Protocol Number: GD20210609). The isolation of BMDMs from C57BL/6 J mice (6–10 weeks) was carried out based on previous procedures^48^. BMDMs were derived from bone marrow cells, which were collected by flushing femur and tibia of C57BL/6 mice, and cultured with RPMI-1640 (Sigma, USA) contained 20% fetal bovine serum (FBS) (BioInd, Kibbutz, Israel), 1% penicillin/streptomycin and 20 ng mL^−1^ of macrophage colony-stimulating factor (M-CSF) (Protein-tech, Chicago, USA). Fresh medium was changed regularly until 80–90% confluent monolayers were obtained.

The following study protocol was approved by the Medical Ethical Committee of School of Stomatology, Shandong University (Protocol Number: GR20210323). The collection and manipulation of human tissue was performed in compliance with all relevant ethical regulations, including the Declaration of Helsinki, and all samples were obtained with written informed consents from the participants. The isolation of hPDLSCs was carried out based on our previously reported procedures^44,49^. Briefly, periodontal ligament tissue was obtained from healthy donors of extracted premolars and third molars aged 12 to 28 years. Periodontal ligament tissue was scraped from the middle third of the root surface and cut into small pieces. Digestion was performed for 40 min at 37 °C in a solution containing collagenase I (3 mg mL^−1^) and dispase II (4 mg mL^−1^). The obtained hPDLSCs were cultured in DMEM supplemented with 10% FBS. hPDLSCs at passages 4–6 were used for the following experiments. HUVECs were commercially purchased (ScienCell, San Diego, USA) and seeded in supplemented endothelial culture medium (ScienCell). All cells were cultured at 37 °C in a 5% CO_2_ incubator with a humidified atmosphere.

The scaffolds were cut into 24 mm in diameter for 6-well culture plate or 14 mm for 24-well plate. The scaffold membranes were soaked in 75% ethanol for 2 h, after that these scaffolds were rinsed with phosphate buffer saline (PBS) solution to remove residual ethanol. Then, the scaffolds were dried under sterile conditions and soaked in α-minimum essential medium (α-MEM) for 24 h. BMDMs, hPDLSCs, and HUVECs were seeded on top of the scaffolds respectively for subsequent experiments.

### Cell viability evaluation

The cytotoxicity of the PLAM-MPN and pure PLAM scaffolds was examined by a LIVE/DEAD viability (Invitrogen, CA, USA)/cytotoxicity kit (Dojindo Laboratories, Tokyo, Japan). hPDLSCs were seeded on different scafollds at a density of 5 × 10^4^ cells/well and incubated for 1, 2, and 3 d. At the end of each time period, the medium was discarded. The scaffolds were rinsed gently thrice with PBS. Next, the staining working solution were prepared according to the instructions: 5 μL calcein-am solution and 10 μL propidium iodide (PI) solution were respectively added to 5 mL PBS at room temperature for 30 min for the live/dead assay. Finally, the different samples were observed under the microscope and photographed. Cell viability and proliferation on different substrates were evaluated by CCK-8 after cultivation for 1, 2, and 3 d.

### hPDLSCs morphology on the scaffolds

First, the growth state of hPDLSCs on the scaffolds was observed by confocal laser scanning microscopy (CLSM, LSM880). In order to identify the porous structure of the scaffold, the red rhodamine B fluorescent dye was added to the PLAM-B, PLAM-P, PLAM-1, PLAM-5, PLAM-10 surface. All the following experimental operations were performed in the dark. After 48 h culture, the medium was discarded. The scaffolds were rinsed gently thrice with PBS. Then, cells were fixed with 4% paraformaldehyde for 10 min at room temperature. After washing with PBS, the cells were stained with DAPI for 5 min. Finally, after washing again with PBS, the growth state and distribution of cells in the stained porous scaffolds were observed under CLSM. The morphology of hPDLSCs on PLAM-10 scaffold was characterized under SEM after dehydration by gradient alcohol.

### In vitro immunoregulation assay

The identity of the obtained BMDMs was tested first by flow cytometry (Accuri-C6, BD Biosciences, San Diego, USA). The detected maker included the PE Anti-F4/80 (1:10, ab105156, Abcam) and FITC anti-mouse/human CD11b (1:200, 101205, Biolegend). After being passaged, the BMDMs were seeded onto the barrier membrane at a density of 2 × 10^5^ cells/well. After culturing for 12 h, the conditioned medium was replaced. Among them, in all groups to detect the M1 polarization, the medium was DMEM containing 100 ng mL^−1^ lipopolysaccharide (LPS) + 20 ng mL^−1^ interferon γ (IFN-γ) in 10% FBS, and the stimulation time was 12 h. The groups to detect the M2 polarization, except that the medium of the “NC + IL-4” group was 20 ng mL^−1^ IL-4 + 20 ng mL^−1^ IL-10 in DMEM with 10% FBS, the rest medium in the group was DMEM with 10% FBS alone, and the stimulation time was 48 h. Next, phenotypic characterization of BMDMs induced by the different scaffolds was performed by flow cytometry. Stimulated BMDMs were incubated with allophycocyanin (APC)-conjugated anti-iNOS (1:333, 17-5920-82, Invitrogen) and anti-CD206 (MMR) (1:40, 141708, Biolegend).

The gene expression levels of tumor necrosis factor (TNF-ɑ), inducible nitric oxide synthase (iNOS), arginase-1 (Arg-1), and CD206 were measured by quantitative reverse transcription polymerase chain reaction (qRT-PCR) to assess the immunoregulation effects of the PLAM-MPN scaffolds on BMDMs, and glyceraldehyde-3-phosphate dehydrogenase (GAPDH) primer was selected as the housekeeping gene and the sequences of the primers were provided in Supplementary Table 1.

In addition, for fluorescence analysis, the samples were fixed with 4% paraformaldehyde for 10 min, and then 10% goat serum (ab7481, Abcam) was added and incubated at 37 °C for 1 h. Subsequently, the cells were incubated with iNOS antibody (1:300, ab49999, Abcam) and CD206 antibody (1:300, ab64693, Abcam) overnight, respectively. The cells were further incubated with CoraLite 488-conjugated goat anti-rabbit secondary antibody (1:800, SA00013-2, Protein-tech) and then counterstained with DAPI (ab104139, Abcam) for 5 min. The images were captured with the fluorescence microscope (Olympus).

### In vitro vascularization assay

After the HUVECs were cultured with different scaffolds respectively for 14 d, the tubule formation assay was performed according to the manufacturer’s instructions. The Matrigel were placed at 4 °C overnight and thaw and the 48-well culture plate and pipette tips were pre-cooled at −20 °C for 1 h in advance. Then, the thawed Matrigel was spread evenly on the pre-cooled 48-well plate (100 μL/well), placed at 37 °C for 30 min to solidify the Matrigel. While waiting for Matrigel to solidify, cells grown on negative control (NC), PLAM, and PLAM-MPN scaffolds were collected by trypsinization and washed twice with PBS. The cells were then resuspended at a density of 5.5 × 10^5 ^mL^−1^. Finally, 100 μL of cell suspension was added to each well of a 48-well plate. After 8 h of incubation, the samples were observed and photographed under microscope. Meanwhile, the relative genes (vascular endothelial growth factor (VEGF), stem cell factor (SCF) and hypoxia-inducible factor (HIF)) expression levels of angiogenic differentiation were analyzed by qRT-PCR. GAPDH primer was selected as the housekeeping gene and the sequences of the primers are provided in Supplementary Table 1.

### In vitro stem cell recruitment assay

The hPDLSCs were digested and adjusted to 5 × 10^5 ^mL^−1^ in α-MEM supplemented with 0.1% FBS, and 200 μL of the cell suspension were inoculated to the upper side of the migration chamber. The scaffolds were then placed in the lower side and soaked in 500 μL of α-MEM containing 0.1% FBS. The NC group was also cultured in α-MEM with 0.1% FBS, and the PC group was cultured with α-MEM with 10% FBS. After the cells incubated at 37 °C for 20 h, the noninvasive cells on the upper side were removed by a cotton swab. Finally, the cells were fixed with 4% paraformaldehyde for 30 min, washed with PBS three times, stained with 0.1% crystal violet for 20 min and washed with PBS. The samples were observed under an optical microscope (Olympus, Tokyo, Japan).

### In vitro hPDLSCs osteogenesis assay

The hPDLSCs were seeded on different scaffolds at a density of 1.5 × 10^5^ cells/well. After the cell adhesion, the medium was replaced with osteogenic induction medium. After 21 d, the samples were rinsed gently with PBS to avoid wrapping the cells on the culture plate. Then the cells were fixed with 4% paraformaldehyde for 30 min. After being rinsed, an appropriate amount of Alizarin Red staining solution was added to each well for 30 min. A digital camera was used to collect the optical image of the overall macroscopic effect of the orifice plate, and the microscopic image of the mineralized nodules was observed under a microscope and photographed. Next, 1 mL of 10% cetylpyridinium chloride (CPC) solution was added to each well of the 6-well plate. After the mineralized nodules were dissolved for 30 min at room temperature, 100 μL of the solution were pipetted into a 96-well plate. Finally, the OD value was measured and analyzed at 562 nm. The relative genes (alkaline phosphatase (ALP), runt-related transcription factor 2 (Runx2) and osteopontin (OPN)) expression levels of osteogenic differentiation were analyzed by qRT-PCR. GAPDH primer was selected as the housekeeping gene and the sequences of the primers were provided in Supplementary Table 1.

### In vivo periodontal bone regeneration assay

Animal studies were approved by the Ethics Committee of Stomatological Hospital of Shandong University (Protocol Number: GD20190901). The animal experimental protocols were performed according to the Animal Research: Reporting of In Vivo Experiments (ARRIVE) guidelines. Wistar rats (male, 7 w, 220 ± 20 g, Chales River, Beijing, China) were used in this study (24 rats for each group, 72 rats in total). they were stored in the animal center at the Shandong University following a 12 + 12 hours dark/light cycle with a fasten 20–25 °C ambient temperature, food (provided and water were autoclave-sterilized prior to administration. The mice were free to access food and water prior and after experiments.

According to the previous published articles^44,50^, the operation was performed under pentobarbital sodium anesthesia (40 mg kg^−1^ body weight). In brief, bilateral periodontal bone defects (5 × 4 × 1 mm^3^, L × H × D) were created with a dental drill, and the defect area was located approximately 1 mm posterior to the anterior border of the mandible and 1 mm below the superior border of the mandible. All rats with defects were numbered and randomly divided into 3 groups, in which different scaffolds were implanted into the defects: (1) no treatment (NC), (2) PLAM, and (3) PLAM-10. The investigator was blinded to animal groupings. After surgery, the animals were intramuscularly injected with penicillin (400,000 U mL^−1^) for three successive days. At 1, 2, 4, and 8 w, the rats were euthanized by injecting excessive pentobarbital anesthesia, then the mandible specimens of the rats were collected and fixed with 4% paraformaldehyde by cardiac perfusion for the following experiments.

### Micro-computed tomography assay

To estimate and compare bone regeneration of defect site in each group, micro-computed tomography (Micro-CT) (PerkinElmer, MA, USA) analysis was applied. Quantitatively, the parameters including the bone volume/total volume (BV/TV), bone surface/total volume (BS/TV), trabecular bone thickness (Tb.Th), trabecular bone separation (Tb.Sp) were analyzed and calculated to assess bone regeneration. The implanted scaffolds were taken out at 8 w post-surgery, and the cell growth on both surfaces of the barrier membrane was observed by SEM.

### Immunofluorescence assay

Decalcification of the specimens was performed with 10% disodium ethylenediaminetetraacetic acid (EDTA-Na_2_, Solarbio Beijing, China) at 4 °C for 30 d. Then, decalcified tissue embedded into paraffin wax was sliced into 5 μm sections. The sections were baked for 1 h at 60 °C and antigen repair was performed with trypsin antigen retrieval solution (BL333A, Biosharp, China). After blocking with goat serum for 2 h, the sections were incubated with primary antibodies overnight at 4 °C, then washed three times for 5 min. Finally, they were sealed with antifade mounting medium with DAPI (BL739A, Biosharp, China). In order to detect the bone regeneration, phenotype switching of macrophages, and angiogenesis in vivo, the tissue sections were incubated with primary antibodies anti-ALP (1:500, ab65834, Abcam), anti-Runx2 (1:500, ab76956, Abcam), anti-CD68 (1:250, ab955, Abcam)/anti-iNOS (1:200, ab15323, Abcam), anti-CD68/anti-CD206(1:200, ab64693, Abcam) and anti-CD31 (1:300, ab28364, Abcam). The fluorescent slides were finally covered by the cover glass using mounting medium containing DAPI. The fluorescent images were obtained with the fluorescence microscope, the fluorescence intensity of ALP and the number of Runx2^+^ cells, CD206^+^/CD68^+^ cells, iNOS^+^/CD68^+^ cells and CD31^+^ cells in the defects were counted and measured by Image J software.

### Statistical analysis

All values are reported as the mean ± standard deviation (SD). Comparisons of three or more groups were compared by assessing one-way or two-way analysis of variance (ANOVA) with Tukey’s HSD post hoc test using GraphPad Prism software (Version 6, MacKiev Software, Boston, MA, USA). Differences were considered statistically significant at *P < 0.05, **P < 0.01, ***P < 0.001.

### Reporting summary

Further information on research design is available in the Nature Research Reporting Summary linked to this article.

## Supplementary information

Supplementary information - Janus Porous Polylactic Acid Membranes with Versatile Metal-Phenolic Interface for Biomimetic Periodontal Bone Regeneration

Reporting Summary

## Data Availability

The data that support the findings of this study are available from the corresponding author on request.
