# Supplementary material for: Janus porous polylactic acid membranes with versatile metal–phenolic interface for biomimetic periodontal bone regeneration
Source: NPJ Regen Med. 2023 Jun 3;8:28. doi: 10.1038/s41536-023-00305-3 (PMC10239453; doi:10.1038/s41536-023-00305-3)
Supplement: Supplementary file 1 — Supplementary information - Janus Porous Polylactic Acid Membranes with Versatile Metal-Phenolic Interface for Biomimetic Periodontal Bone Regeneration [file 41536_2023_305_MOESM1_ESM.pdf]

Supplementary Information for

Janus Porous Polylactic Acid Membranes with Versatile Metal-Phenolic Interface for Biomimetic Periodontal Bone Regeneration

Yaping Zhang [1, 2, #], Yi Chen [1, #], Tian Ding [1], Yandi Zhang [1], Daiwei Yang [1], Yajun Zhao [1], Jin Liu [1], Baojin Ma [1], Alberto Bianco [3], Shaohua Ge [1, *], Jianhua Li [1, *]

1. Department of Biomaterials, School and Hospital of Stomatology, Cheeloo College of Medicine, Shandong University & Shandong Key Laboratory of Oral Tissue Regeneration & Shandong Engineering Laboratory for Dental Materials and Oral Tissue Regeneration & Shandong Provincial Clinical Research Center for Oral Diseases, Jinan 250012, China

2. Department of Orthodontics, The First Affiliated Hospital of Zhengzhou University, (Stomatological Hospital of Henan Province), Zhengzhou 450052, China

3. CNRS, Immunology, Immunopathology and Therapeutic Chemistry, UPR 3572, University of Strasbourg, ISIS, 67000 Strasbourg, France

*Corresponding Authors:

Jianhua Li

E-mail: jianhua.li@sdu.edu.cn

Shaohua Ge

E-mail: shaohuage@sdu.edu.cn

[#] First authors: These authors contributed equally to this work.

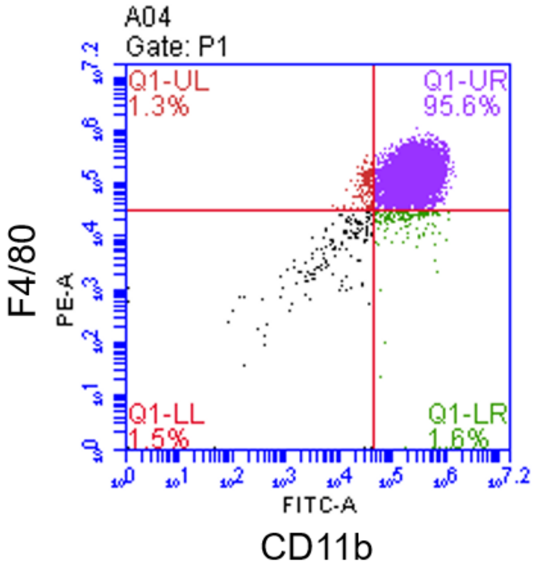

Supplementary Figure 1. Purity identification of primary BMDMs.

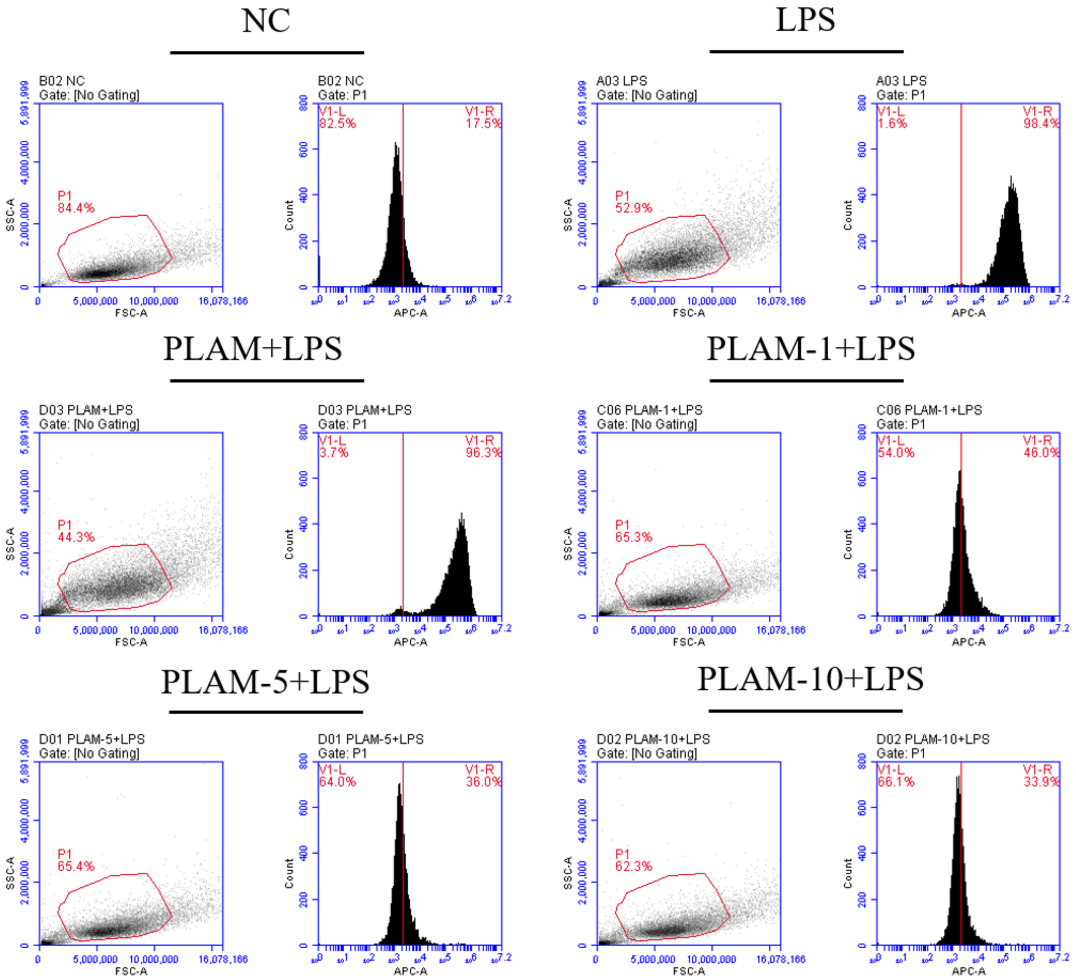

Supplementary Figure 2. All FACS sequential gating for M1-type macrophages analysis, the APC gating panel correspond to the Figure 6b in the manuscript.

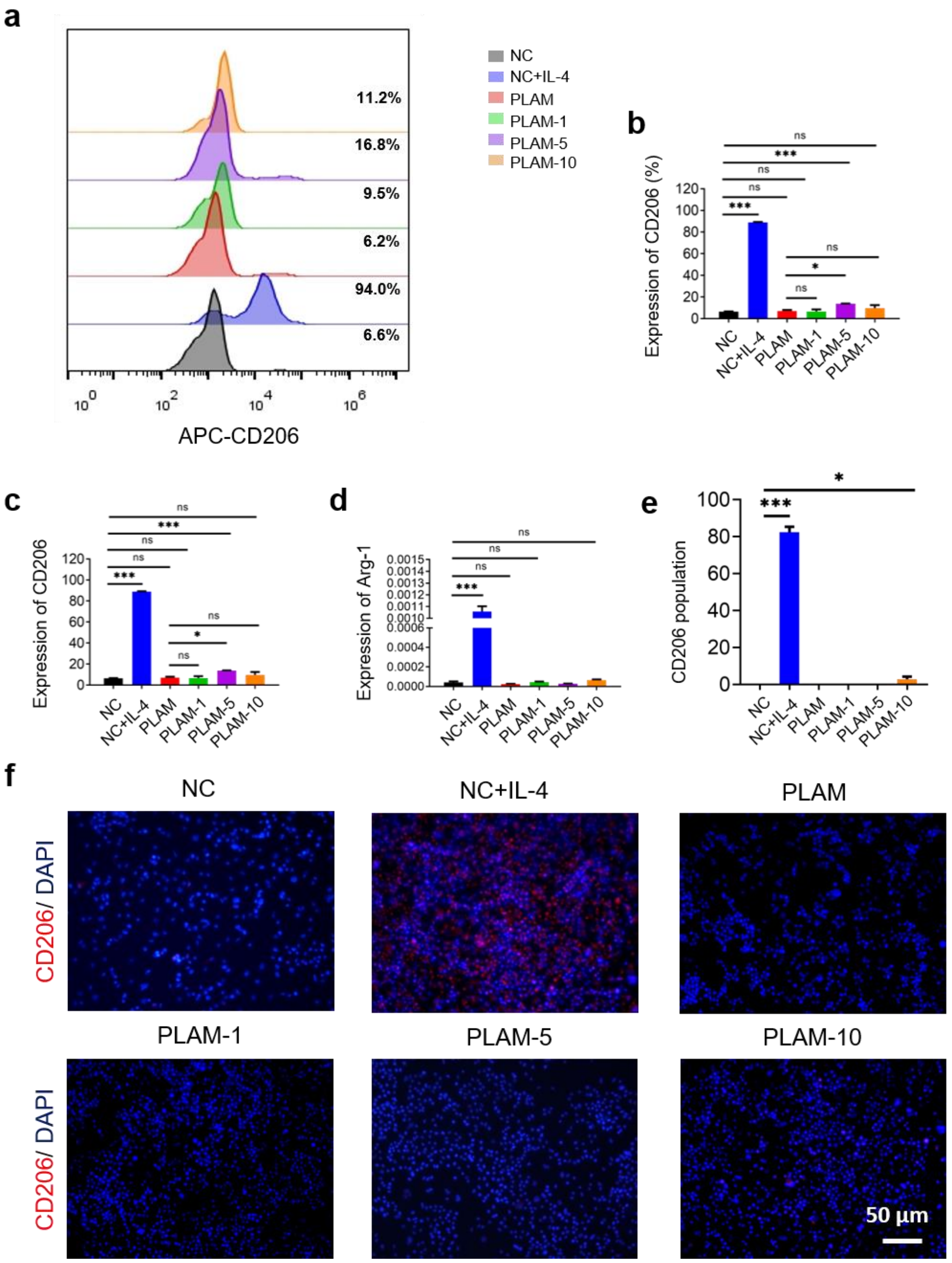

Supplementary Figure 3. Immune regulation of macrophages on the scaffolds. a, b) Flow cytometry assay and quantitative analysis of CD206 in BMDMs (n = 3 independent experiments). c, d) Relative mRNA expressions of CD206 and Arg-1 in BMDMs (n = 3 independent experiments). e, f) Quantitative analysis and representative immunofluorescent staining images of CD206 in BMDMs (blue represents DAPI, red represents CD206, n=3 independent experiments, four random fields in each sample,

scale bar=50 μm). All data are shown as mean ± SD, ns = statistical nonsignificance, $**P < 0.01$ and $***P < 0.001$.

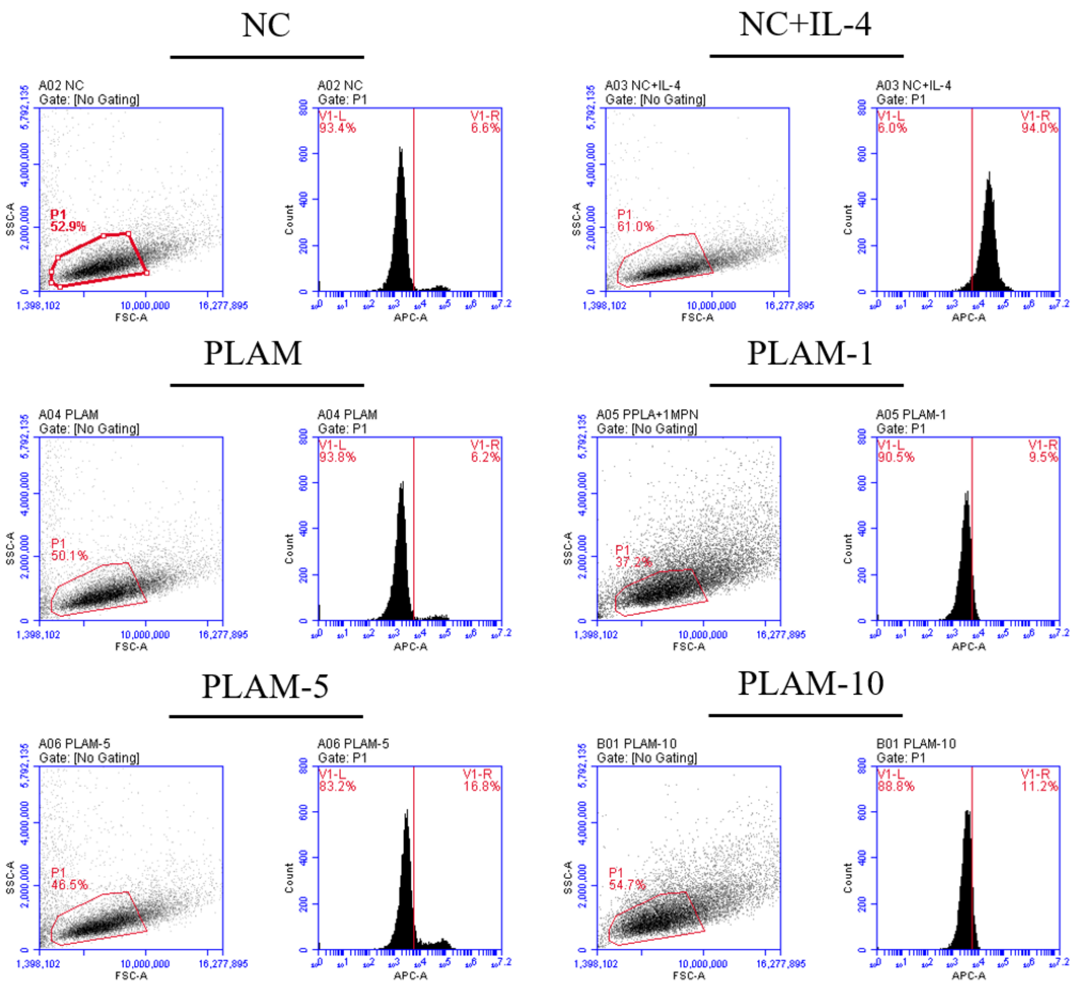

Supplementary Figure 4. All FACS sequential gating for M2-type macrophages analysis, the APC gating panel correspond to the Figure 6b in the manuscript.

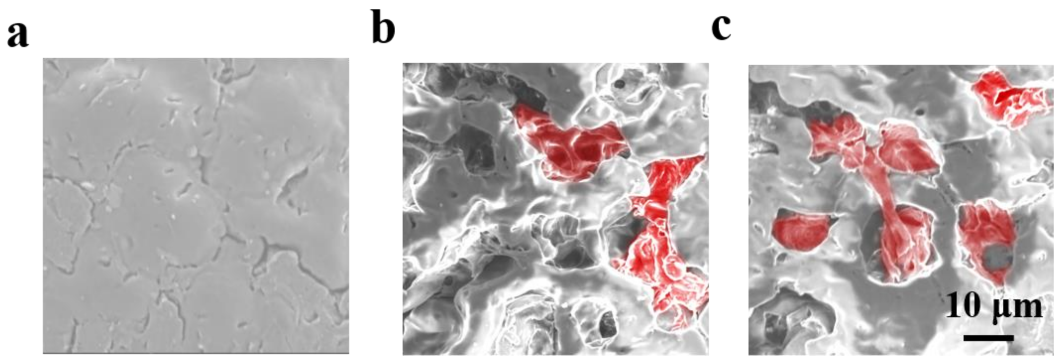

Supplementary Figure 5. Evaluation of the barrier function of PLAM-MPN scaffolds. SEM image of the scaffolds' surface near the defect at 8 w after surgery. a) PLAM-B surface. b) PLAM-P. c) PLAM-10 surface.

Supplementary Table 1 Primer sequences were used for PCR in the present study.

**Gene**	**Forward primer (5'~3')**	**Reverse primer (5'~3')**
**GAPDH**	TGTCTCCTGCGACTTCAACA	GGTGGTCCAGGGTTTCTTACT
**iNOS**	CCTGCTTTGTGCGAAGTGTC	CCCAAACACCAAGCTCATGC
**TNF-α**	CGGGCAGGTCTACTTTGGAG	ACCCTGAGCCATAATCCCCT
**Arg-1**	TGTCCCTAATGACAGCTCCTT	GCATCCACCCAAATGACACAT
**CD206**	ACCTGGGGACCTGGTTGTAT	CTCGCGTCCAATAGCTGAAC
**VEGF**	CTTGCAGATGTGACAAGCCG	GTCGATGGTGATGGTGTGGT
**HIF**	GGCAGCAACGACACAGAAAC	TTTTCGTTGGGTGAGGGGAG
**SCF**	GACCTTGTGGAGTGCGTGAA	CTGGGTTCTGGGCTCTTGAAT
**ALP**	GGCGGTGAACGAGAGAATGT	GGACGTAGTTCTGCTCGTGG
**RUNX2**	GGAGTGGACGAGGCAAGAGT	AGGCGGTCAGAGAACAAACT
**OPN**	GCCGTGGGAAGGACAGTTAT	ATCTGGACTGCTTGTGGCTG
