# Supplementary material for: Janus porous polylactic acid membranes with versatile metal–phenolic interface for biomimetic periodontal bone regeneration
Source: NPJ Regen Med. 2023 Jun 3;8:28. doi: 10.1038/s41536-023-00305-3 (PMC10239453; doi:10.1038/s41536-023-00305-3)
Supplement: Supplementary file 2 — Reporting Summary [file 41536_2023_305_MOESM2_ESM.pdf]

nature portfolio

Corresponding author(s): Jianhua Li and Shaohua Ge

Last updated by author(s): Mar 13, 2023

# Reporting Summary

Nature Portfolio wishes to improve the reproducibility of the work that we publish. This form provides structure for consistency and transparency in reporting. For further information on Nature Portfolio policies, see our Editorial Policies and the Editorial Policy Checklist.

## Statistics

For all statistical analyses, confirm that the following items are present in the figure legend, table legend, main text, or Methods section.

| n/a | Confirmed | |
|---|---|---|
| ☐ | ☒ | The exact sample size (*n*) for each experimental group/condition, given as a discrete number and unit of measurement |
| ☐ | ☒ | A statement on whether measurements were taken from distinct samples or whether the same sample was measured repeatedly |
| ☒ | ☐ | The statistical test(s) used AND whether they are one- or two-sided<br>*Only common tests should be described solely by name; describe more complex techniques in the Methods section.* |
| ☐ | ☒ | A description of all covariates tested |
| ☒ | ☐ | A description of any assumptions or corrections, such as tests of normality and adjustment for multiple comparisons |
| ☐ | ☒ | A full description of the statistical parameters including central tendency (e.g. means) or other basic estimates (e.g. regression coefficient) AND variation (e.g. standard deviation) or associated estimates of uncertainty (e.g. confidence intervals) |
| ☐ | ☒ | For null hypothesis testing, the test statistic (e.g. *F*, *t*, *r*) with confidence intervals, effect sizes, degrees of freedom and *P* value noted<br>*Give P values as exact values whenever suitable.* |
| ☒ | ☐ | For Bayesian analysis, information on the choice of priors and Markov chain Monte Carlo settings |
| ☒ | ☐ | For hierarchical and complex designs, identification of the appropriate level for tests and full reporting of outcomes |
| ☒ | ☐ | Estimates of effect sizes (e.g. Cohen's *d*, Pearson's *r*), indicating how they were calculated |

*Our web collection on statistics for biologists contains articles on many of the points above.*

## Software and code

Policy information about availability of computer code

| | |
|---|---|
| Data collection | *Provide a description of all commercial, open source and custom code used to collect the data in this study, specifying the version used OR state that no software was used.* |
| Data analysis | *Provide a description of all commercial, open source and custom code used to analyse the data in this study, specifying the version used OR state that no software was used.* |

For manuscripts utilizing custom algorithms or software that are central to the research but not yet described in published literature, software must be made available to editors and reviewers. We strongly encourage code deposition in a community repository (e.g. GitHub). See the Nature Portfolio guidelines for submitting code & software for further information.

## Data

Policy information about availability of data

All manuscripts must include a data availability statement. This statement should provide the following information, where applicable:

- Accession codes, unique identifiers, or web links for publicly available datasets
- A description of any restrictions on data availability
- For clinical datasets or third party data, please ensure that the statement adheres to our policy

The data that support the findings of this study are available from the corresponding author on request.

## Human research participants

Policy information about studies involving human research participants and Sex and Gender in Research.

| | |
|---|---|
| Reporting on sex and gender | The influence of sex and gender was not considered in the experimental design of this study. All samples who met the inclusion criteria were selected according to the requirements of periodontal ligament tissue required for periodontal ligament stem cell isolation and culture. |
| Population characteristics | Healthy volunteers aged 12 to 28 years who needed extraction of wisdom teeth or orthodontic teeth without caries or periodontal disease. |
| Recruitment | The patients who met the inclusion criteria were selected from the dental surgery clinic and signed the informed consent form before the subsequent experiment was carried outThe patients who met the inclusion criteria were selected from the dental surgery clinic and signed the informed consent form before the subsequent experiment was carried out. |
| Ethics oversight | Medical Ethical Committee of School of Stomatology, Shandong University (Protocol Number: GR20210323). |

Note that full information on the approval of the study protocol must also be provided in the manuscript.

# Field-specific reporting

Please select the one below that is the best fit for your research. If you are not sure, read the appropriate sections before making your selection.

☒ Life sciences ☐ Behavioural & social sciences ☐ Ecological, evolutionary & environmental sciences

For a reference copy of the document with all sections, see nature.com/documents/nr-reporting-summary-flat.pdf

# Life sciences study design

All studies must disclose on these points even when the disclosure is negative.

| | |
|---|---|
| Sample size | All the experiments were performed on replicate samples with multiple field of views taken for each experiment as described in Methods section. |
| Data exclusions | No data was excluded. |
| Replication | All attempts at replication were successful. |
| Randomization | In order to reduce the differences between individuals and ensure the authenticity of the final experimental data, a completely randomized design was adopted for allocation. Animals were numbered one by one and then grouped using a random number table. |
| Blinding | Investigators were unaware of group assignments when analyzing the data. |

# Reporting for specific materials, systems and methods

We require information from authors about some types of materials, experimental systems and methods used in many studies. Here, indicate whether each material, system or method listed is relevant to your study. If you are not sure if a list item applies to your research, read the appropriate section before selecting a response.

### Materials & experimental systems

| n/a | Involved in the study |
|---|---|
| ☐ | ☒ Antibodies |
| ☒ | ☐ Eukaryotic cell lines |
| ☒ | ☐ Palaeontology and archaeology |
| ☐ | ☒ Animals and other organisms |
| ☒ | ☐ Clinical data |
| ☒ | ☐ Dual use research of concern |

### Methods

| n/a | Involved in the study |
|---|---|
| ☒ | ☐ ChIP-seq |
| ☐ | ☒ Flow cytometry |
| ☒ | ☐ MRI-based neuroimaging |

## Antibodies

| | |
|---|---|
| Antibodies used | PE Anti-F4/80 (1:10, ab105156, Abcam), FITC anti-mouse/human CD11b (1:200, 101205, Biolegend), allophycocyanin (APC)- |

| | |
|---|---|
| Antibodies used | conjugated anti-iNOS (1:333, 17-5920-82, Invitrogen), anti-CD206 (MMR) (1:40, 141708, Biolegend), iNOS antibody (1:300, ab49999, Abcam), CD206 antibody (1:300, ab64693, Abcam), CoraLite 488-conjugated goat anti-rabbit secondary antibody (1:800, SA00013-2, Protein-tech), anti-ALP (1:500, ab65834, Abcam), anti-Runx2 (1:500, ab76956, Abcam), anti-CD68 (1:250, ab955, Abcam), anti-iNOS (1:200, ab15323, Abcam), anti-CD206(1:200, ab64693, Abcam), anti-CD31 (1:300, ab28364, Abcam). |
| Validation | PE Anti-F4/80 (Abcam, ab105156),Host Species: Mouse; Isotype: IgG2b; Suitable for: Flow Cyt.<br>FITC anti-mouse/human CD11b (Biolegend, 101205).Host Species: Rat; Isotype: IgG2a, kappa; Suitable for: Flow Cytometry (FCM).<br>Allophycocyanin (APC)-conjugated anti-iNOS (Invitrogen, 17-5920-82), Host Species: Rat; Isotype: IgG2a, kappa; Suitable for: Flow Cytometry (FCM).<br>APC anti-mouse CD206 (MMR) antibody (Biolegend, 141708)., Host Species: Rat; Isotype: IgG2a, kappa; Suitable for: FCM, intracellular staining for Flow Cytometry.<br>iNOS antibody (Abcam, ab49999), Host Species: Mouse; Isotype: IgG1; Suitable for: ICC/IF, WB.<br>CD206 antibody(Abcam, ab64693), Host Species: Rabbit; Isotype: IgG; Suitable for: IHC-P, WB, ICC/IF.<br>anti-ALP (Abcam, ab65834), Host Species: Rabbit; Isotype: IgG; Suitable for: WB, IHC-P.<br>anti-Runx2 (Abcam, ab76956), Host Species: Mouse; Isotype: IgG2a; Suitable for: ICC/IF, Flow Cyt, WB.<br>anti-CD68 (Abcam, ab955), Host Species: Mouse; Suitable for: ICC/IF, WB, IHC-P.<br>anti-iNOS (Abcam, ab15323), Host Species: Rabbit; Isotype: IgG; Suitable for: IHC-P, WB.<br>anti-CD206(Abcam, ab64693,) Host Species: Rabbit; Isotype: IgG; Suitable for: IHC-P, WB, ICC/IF.<br>anti-CD31 (Abcam, ab28364). Host Species: Rabbit; Isotype: IgG; Suitable for: IHC-P. |

## Animals and other research organisms

Policy information about studies involving animals; ARRIVE guidelines recommended for reporting animal research, and Sex and Gender in Research

| | |
|---|---|
| Laboratory animals | Wistar rats (male, 7 w, 220±20 g, Chales River, Beijing, China) |
| Wild animals | N/A |
| Reporting on sex | N/A |
| Field-collected samples | N/A |
| Ethics oversight | Animal studies were approved by the Ethics Committee of Stomatological Hospital of Shandong University (Protocol Number: GD20190901). |

Note that full information on the approval of the study protocol must also be provided in the manuscript.

## Flow Cytometry

### Plots

Confirm that:

☒ The axis labels state the marker and fluorochrome used (e.g. CD4-FITC).

☒ The axis scales are clearly visible. Include numbers along axes only for bottom left plot of group (a 'group' is an analysis of identical markers).

☒ All plots are contour plots with outliers or pseudocolor plots.

☒ A numerical value for number of cells or percentage (with statistics) is provided.

### Methodology

| | |
|---|---|
| Sample preparation | The rat bone marrow-derived macrophages (BMDMs) were isolated from rat and cultured in dulbecco's modified eagle medium (DMEM) medium contained 20% fetal bovine serum (FBS) (BioInd, Kibbutz, Israel) and 1% penicillin/streptomycin and 20 ng mL−1 of macrophage colony-stimulating factor (M-CSF) (Protein-tech, Chicago, USA). Fresh medium was changed regularly until 80-90% confluent monolayers were obtained. |
| Instrument | Accuri-C6, BD Biosciences, San Diego, USA |
| Software | Flow Jo |
| Cell population abundance | The purity of BMDMs was determined based on quantification of cell populations expressing both antibodies, CD11b and F4/80. |

Gating strategy

In the macrophage polarization experiment, according to the FSC/sSC circle gate, the cell activity was better, about 60.1%, from the selected gate cells, we further detected the antibody expression. As shown in Figure 3b, we set up an isotype negative control, which represents the non-specific fluorescence of the cells themselves. This negative control was used to exclude the influence of fluorescein. As shown in the figure, the iNOS expression rate of the negative control group was 17.4%. At the same time, we set up a positive control group with LPS+IFN-r. As can be seen from the figure, the expression rate of iNOS in the positive control group was 98.4%.

☒ Tick this box to confirm that a figure exemplifying the gating strategy is provided in the Supplementary Information.
